# Supplementary material for: Preventive and curative dental services utilization among children aged 12 years and younger in Tehran, Iran, based on the Andersen behavioral model: A generalized structural equation modeling
Source: PLoS One. 2025 Jan 16;20(1):e0312043. doi: 10.1371/journal.pone.0312043 (PMC11737785; doi:10.1371/journal.pone.0312043)
Supplement: S1 File — (DOCX) [file pone.0312043.s001.docx]

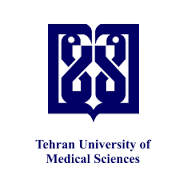


This questionnaire is designed for data collection related to PhD thesis entitled “Evaluation of oral health services utilization and its determinants, (based on Anderson model), and assessment of inequality in oral health (by decomposition analysis) among eighteen-year-old and younger inhabitants, in Tehran, 2023”, approved by Tehran university of Medical Science (ethical code: IR.TUMS.DENTISTRY.REC.1401.094)

We are thankful if you kindly answer the questions concisely, your name does not need to be disclosed. The interviews will conduct in a confidential environment. You can cease participation or withdraw from the study at any time.

Are you willing to participate in this study?

**A) Demographic characteristics of head of household:**

1) Age ……... 2) Gender ……...

3) Which district do you live in? ……..

4) Size of household (number of individuals living in your house):

5) What is your education?

1. Literate and less than the diploma
2. Diploma
3. Associate and bachelor
4. Master and more

6) How much is your family's average monthly income?

1. 5 million IRR and less
2. 5 to 10 million IRR
3. 10 to 15 million IRR
4. More than 15 million IRR

**B) Demographic characteristics of the children:**

1) Age ……. 2) Gender ……

3) Does your child have basic insurance?

1) Yes 2) No 3) I don’t know

4) Does your child have dental insurance?

1) Yes 2) No 3) I don’t know

**C) Oral Health information of the children:**

1) How would you describe your children's oral health?

1. Very poor
2. Poor
3. Moderate
4. Good
5. Very good
6. Excellent

2) How often does your child brush their teeth?

1. Irregularly
2. Once a day
3. More than once a day

3)How often does your child consume snacks and sweet beverages?

1. Three times a day or more
2. Once or twice a day
3. Every week
4. Every month or less

4) How old was your child old in her/his first dental visit?

……… She/he does not ever have dental visit

5) Has your child needed a dental visit in the past year?

1) Yes 2) No 3) I don’t know

6) Did your child have a dental visit in the past year?

1) Yes 2) No 3) I don’t know

7) Which dental services did your child receive in the past dental visit?

1. Examination/Consultation
2. Fissure sealant
3. Fluoride therapy
4. Restoration/extraction
5. Dental pain
6. Others

**D) Parent oral health knowledge**

What do you think of the following statements? (Choose only one answer for every sub-question)

If the primary tooth is diseased, it doesn’t need to be treated.

Correct Incorrect I don’t know

Pit and fissure sealant can prevent children from dental caries.

Correct Incorrect I don’t know

Fluoride can’t protect teeth.

Correct Incorrect I don’t know

The first dental visit should be by the eruption of the first teeth.

Correct Incorrect I don’t know

Regular dental visits are necessary for good oral health.

Correct Incorrect I don’t know

Cariogenic bacteria can transfer from the mother to the child.

Correct Incorrect I don’t know
